# Ability of dynamic holography in self-assembled hybrid nanostructured silica films for all-optical switching and multiplexing

**DOI:** 10.1186/s11671-015-0898-z

**Published:** 2015-04-23

**Authors:** German Telbiz, Svitlana Bugaychuk, Eugen Leonenko, Liudmyla Derzhypolska, Vladimir Gnatovskyy, Igor Pryadko

**Affiliations:** L.V. Pisarzhevsky Institute of Physical Chemistry, National Academy of Sciences of Ukraine, Prospect Nauki 31, Kiev, 03128 Ukraine; Institute of Physics, National Academy of Sciences of Ukraine, Prospect Nauki 46, Kiev, 03028 Ukraine; Physical Department, Taras Shevchenko National University, Prospect Glushkova 4, Kiev, 03127 Ukraine

**Keywords:** Sol-gel, Self-assembled, Mesostructured hybrid film, Rhodamine 6G, Optical spectra, Luminescence, Lifetime, Dynamical holography, All-optical switching

## Abstract

The sol-gel method has been employed in the fabrication of easily processable mesostructured films consisting of a nonionic surfactant and silica as the inorganic component. The ability of the occluded Pluronic P123 mesostructures to solubilize guest molecules made these films ideal host matrices for organic dyes and molecular assemblies, possessing substantial nonlinear susceptibilities. These films were explored for use as the photonic layer in all-optical time-to-space converters and proved successful at increasing the optical response of the intercalated dyes to a point that would make these composite films applicable for use as the photonic layer. Recording of a dynamical grating in a single-pulse regime has been obtained. Since the dynamical grating exhibits the fast relaxation time (up to 10 ns), the nonlinear mechanism represents an electronic excitation of the photosensitive molecules. As far as the dye molecules are distributed in nanoporous silica, a model of ‘gas of molecular dye’ may be rightly used in order to consider nonlinear optical properties in the nanostructured hybrid films. We suppose that further improvement of the nonlinear optical nanomaterials may follow on the way to embed additional inclusions, which will not promote the heat accumulation in the host matrix and will lead to effective dissipation of the heat energy.

**PACS:** 78.20.-e; 42.70 -а; 42.79

## Background

All-optical methods of manipulation of light beams play a growing role in relation to increase speed of optical information processing. Nowadays, the current integral optical architectures are able to implement such operations with optical beams as switching, multiplexing-demultiplexing, amplification, logic elements, and image processing [1]. Many types of optical nonlinear phenomena are the subject of a fast growing area of research to fulfill optical switching and multiplexing, such as changing the bandgap structure in photonic crystal waveguides [2], resonance effects in nanocomposite hybrid cells [3,4], the creation of special layouts of silicon-based nanostructures [5,6], plasmon-polariton modes in metal-dielectric samples [7,8], electro-optical effects in waveguides [9], electrostatic attenuation [10], etc. The dynamic holographic method based on light-induced refractive index change during the interaction of beams in a material occupies a special place because it allows to potentially implement almost all currently known functions of manipulation of light [11,12]. Also, the dynamic holography has been successfully used to achieve diverse effects including optical bistability, phase conjugation, all-optical high-contrast switching, spatial switching, optically addressed spatial light modulators, image amplification, and digital image processing. Besides, the dynamic holography can be applied as an efficient method to investigate nonlinearoptical properties in new materials [13,14]. Therefore, the demand for better materials with desired characteristics arises due to increased optical applications and to reach the maximum potential of holographic information storage.

With using the dynamic holography on thin nanostructures films, the spatial switching with high contrast comes naturally owing to two features: (i) multiple diffraction orders appear due to diffraction of writing beams on the induced dynamical grating and (ii) the existence of the diffraction depends on the intensity of writing beams, what is general for nonlinear optical materials. Except for that, the spatial beam multiplexing may be carried out by means of the diffraction of a weak probe beam on a stable phase grating. In this case, the number of the diffraction orders and their intensities are defined by a form of the grating, i.e., its period, the phase modulation depth, and the shape of the profile of the line [15]. The recent growth in network-connected devices has lead to an increasing demand on the telecommunications network to interconnect these devices with the possible lowest data transfer delay. Thus far, the materials that have shown the most promise to be used as the thin-film photonic layer are either the molecular beam epitaxy grown semiconductors or the neat organic dye films [16]. Organic films are certainly a more cost-effective photonic material since they can be readily fabricated at room temperature, using spin or dipcoating techniques. Such films have been shown to have excellent transmission characteristics and optical responses well into the subpicosecond domain. But these films tend to suffer from poor photostability, which limits their use as the photonic layer. The best active layer material would be one that combines high photostability of the semiconductors and the ease of fabrication as well as the transmission characteristics of organic films. A proven method to fabricate such hybrid materials is by incorporating suitable organic dyes into optically inert inorganic matrices. This can be made using several different methods, but the sol-gel method has been the most extensively applied over the years [17,18]. In this method, the fabrication of glass-like materials is passed through the hydrolysis and condensation of the desired metal alkoxide. By far, the most widely fabricated inorganic matrices are silicate based, due to the excellent physical and optical properties of the resultant materials.

The requirement to the material, in which a photonic layer should possess an ultrafast optical response, is the reason why hybrid dye/silica films have tested in this role. Intercalation of dyes within a silica matrix tends to enhance their photophysical properties as a result of isolation within the photochemically stable inorganic micro-environment. In such an environment, the number of deactivation pathways, which are available for return to the ground state after photoexcitation, is substantially reduced for the majority of dyes. Miniature sustainable micro-gratings may be created on the base of hybrid silica nanostructures in combination with the properties of the waveguide that there is an urgent requirement of integral optical circuits.

In the present work, the sol-gel method has been employed in the fabrication of easily processable, high-quality composite films which consisted of a silica framework, and self-assembled organic mesostructures formed by the nonionic surfactant Pluronic P123 [16] served as the host matrix for various organic dyes and quenchers. The Rhodamine 6G (Rh6G) or carbon (SWNT) nanotubes as the guest molecules are self-organized in the structure of mesostructured silica thin films. The material was thoroughly characterized from the structural and spectroscopic points of view. The ability of dynamic holography in ultrafast excited-state dynamics of developed composite films was examined for its potential use as the photonic layer in an all-optical switching and multiplexing device.

## Methods

### Characterization and sol-gel technology of self-assembled mesostructured hybrid dye/silica films

**Figure 1:**
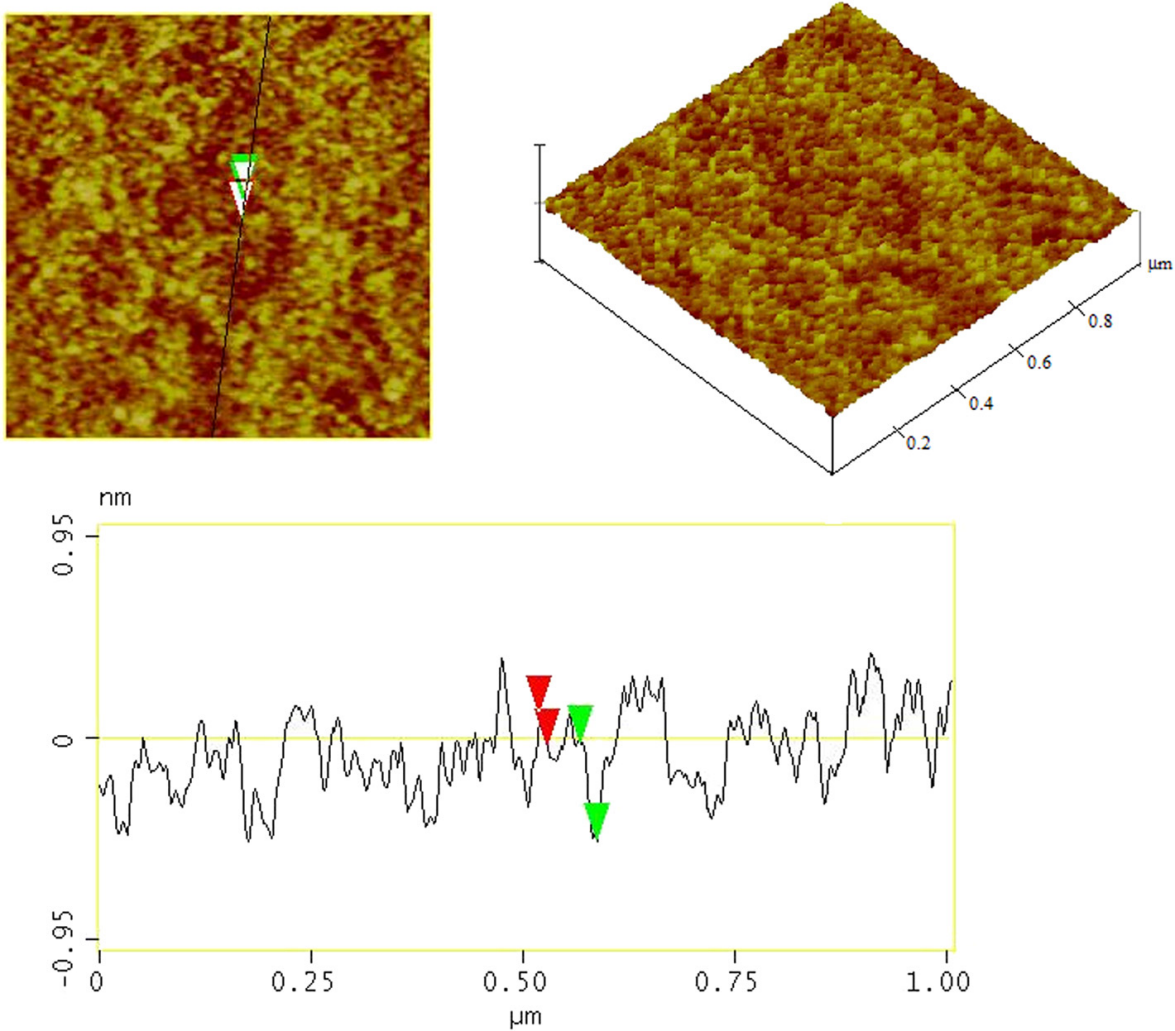
Typical AFM image and roughness characteristics of the hybrid film.

Mesostructured SiO_2_ films were prepared via the template sol-gel pre-doping technique using as precursor material composed of tetraethoxysilane (TEOS), ethanol, distilled water, HCl, and Pluronic 123(P123) (1:8:2:0,5:0,01 molar ratios), and dye or SWNT. This sol composition has proven to give good quality SiO_2_ coatings. In a typical preparation, the TEOS was dissolved in the ethanol using magnetic stirring for 15 min. For preparation of starting composition, 0.003 to 0.72 g of Rhodamine 6G was dissolved in 10 ml sol. The dye concentration was in the range of 6·10^−4^to 1.5·10^−1^ mol/l (SWNT 00.0003 to 0.005 g/ml). This solution was mixed at 60°C for 120 min to form a sol that was used in all of the following operations. The colored films were obtained on glass substrates using a handmade spin and dip coating apparatus and various rotation and withdrawal speeds (1,500 to 2,500 rev/min or 5 to 12 cm/min, respectively). Standard procedure of substrates (cleaned in hot chromic mixture, followed by a rinse with distilled water) was adopted before coating. After that, the coated films were dried at ambient temperature during 48 h at atmospheric air conditions. The coating thickness measured by atomic force microscopy (AFM) was approximately 200 or 800 nm for spin and dipcoating film, respectively. Typical AFM image and the roughness characteristics of the prepared hybrid films are shown on the Figure 1. The main advantages of the pre-doping method with respect to the post-doping one can be homogeneous distribution of the guest molecules due to mixing of the components at a molecular level and the possibility of introducing larger amounts of guest molecules and reducing the spontaneous aggregation of guest molecule self-aggregation.

The optical absorption spectra were measured at room temperature with a Specord PC 210 (Analytic Jena, Thuringia, Germany) spectrophotometer. Steady-state photoluminescence spectra was measured with Hitachi MPF-4 (Hitachi, Ltd., Chiyoda, Tokyo, Japan) and a PerkinElmer LS55 (PerkinElmer, Inc., Waltham, MA, USA) spectrophotometer. Fluorescence spectra and decay kinetics of Rh6G/SiO_2_ films were measured by the Edinburgh Instruments TCSPC fluorescence spectrometer F900 (Edinburgh Instruments Ltd., Livingston, West Lothian, UK) The diode laser EPL-375 emitting 50-ps pulses with 0.15 mW average power was used for the sample excitation [19]. The surface relief and thickness of the films were revealed by atomic force microscopy using NanoScope D 300 (Digital Instruments, Santa Barbara, CA, USA). Optical properties of a doped film were calculated using envelope method proposed by Swanepoel [20].

### The dynamic holographic investigation of the nanocomposite films

We use a typical two-wave mixing scheme [13] to study holographic properties of Rh6G/SiO_2_ nanocomposite films. The beam from pulsed Q-switched double frequency Nd:yttrium aluminum perovskite (YAP) laser (the wavelength is *λ* = 539.8 nm, the duration of pulse is *τ* = 20 ns, the maximum energy in one pulse is *E*_*p*_ = 1 mJ, the one-mode regime) is divided by a splitting plate on two beams of equal intensities. These two beams are converged on the sample by using an adjusting system that has a mirror on the path of the second beam. The coincidence plane of the two beams is on the plane of the film, where the pump beams form a fringe interference pattern. The period of the interference pattern is 5 μm. In the self-diffraction regime, we observe two additional diffraction orders ({+1} and {−1}) aside from main pump beams ({+0} and {−0} diffraction orders) created due to diffraction of the pump beams from the grating induced by them. The intensity in a first diffraction order is detected by fast p-i-n photodiode connected to a two-channel Velleman PCLab2000SE oscilloscope (Velleman, Legen Heirweg, Gavere, Belgium) (1 GS/s, 50 MHz). The diode retardation time does not exceed 0.5 μs. The recording grating is defined also by a weak probe beam from a continuous semiconductor laser (*λ* = 405 nm, the power is *I*_0_ = 50 mW). The second registering photodiode is put to monitor the intensity in the first diffraction order of the probe beam. The retardation time of the diode does not exceed 5 μs.

## Results and discussion

Due to unique photophysical properties, the applications of Rhodamine 6G (Rh6G) have been diverse. In this case, it is only natural that composite films containing this dye be explored for potential use as the photonic layer. However, Rh6G’s relatively long-lived excited state needs to be significantly reduced to allow for optical switching operations in the ultrafast region. In this case, as Rh6G has its strong absorption and fluorescence in the visible region, it is often necessarily taken to minimize the self-assembly aggregation process between individual dye molecules.

We investigate optical and nonlinear optical properties of different hybrid Rh6G/SiO_2_ nanostructured films. The samples are gathered in Table 1. The samples No.1(2) and No.3 have different concentrations of the dye molecules. The samples No.1 and No.2 are prepared by using dipcoating technique (No.1) or spincoating (No.2). The sample No.4 contains inclusions of SWNT in order to investigate the ability to increase the scattering of the accumulated heat in such nanostructured films.

**Table 1:** **The hybrid mesostructure silica films photosensitive at the wavelength 539.8 nm**

**Number of the sample**	**Description**
1	SiO_2_+P123+Rh6G(0.0036) dip
2	SiO_2_+P123+Rh6G(0.0036) spin
3	SiO_2_+P123+Rh6G(0.0018)
4	SiO_2_+P123+Rh6G(0.0036)+SWNT(0.0003)

The concentrations of the Rh6G and the SWNT are pointed out in parentheses in g/ml units. P123 denotes the template molecules in the host mesostructure [21].

**Figure 2:**
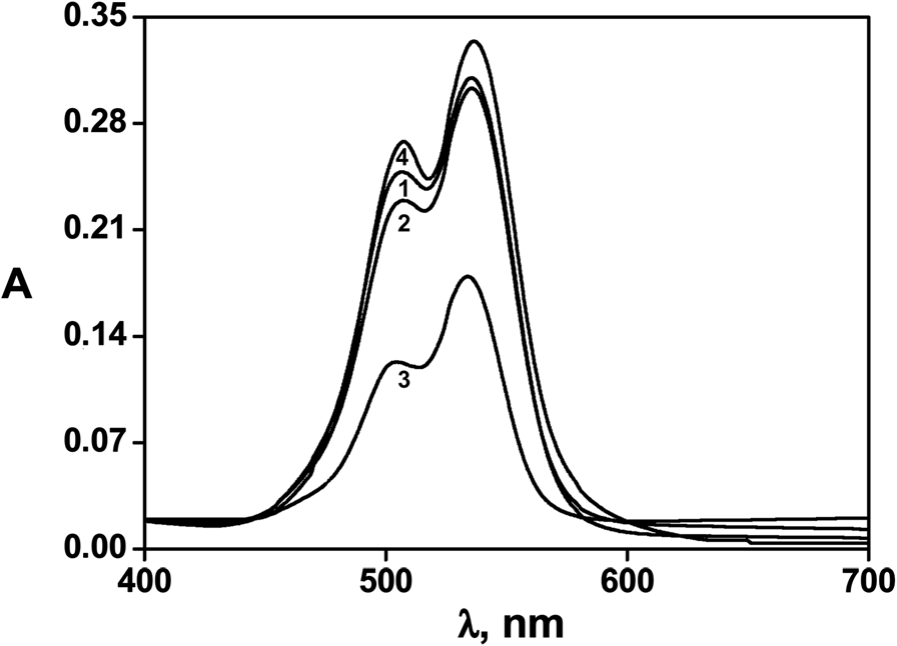
Absorption spectra of hybrid dye/silica nanocomposites. Numbers denote the number of a sample in Table 1.

The absorption spectra of the samples No.1 and No.4 are shown in Figure 2. Spectrum of the Rh6G/SiO_2_ films is almost identical to that in solution, where the main absorption band of Rh6G in visible region corresponds to a transition moment largely parallel to the long axis of the molecule due to π − π* transition. Corresponding absorption maximum at 527 nm and a shoulder at 500 nm are observed in the spectra [18]. But increasing of concentration leads to the intermolecular interaction involved in the formation of the dimer and the intensity of the shoulder at approximately 500 nm becomes much more well-defined and broadened. These changes are most likely most indicative of the formation of a greater number of H-type aggregates, along with the formation of J-type aggregates according to a well-known exciton model.

Spin coating and dip coating are two basic techniques used to produce a thin film on a planar substrate. Both techniques are used to make different coatings and thin films with a wide range of thickness, surface morphology, and tunable microstructure. In the case of dipcoating, structure formation continues more than a minute and aggregation of dye molecules will be spontaneous, while in the case of spincoating, aggregation of dye molecules will be quickly stopped and formation of complex with fragment of Pluronic molecules can take place. We evaluated optical properties of doped films obtained from the same film forming a sol by spin coating and dip coating techniques.

**Figure 3:**
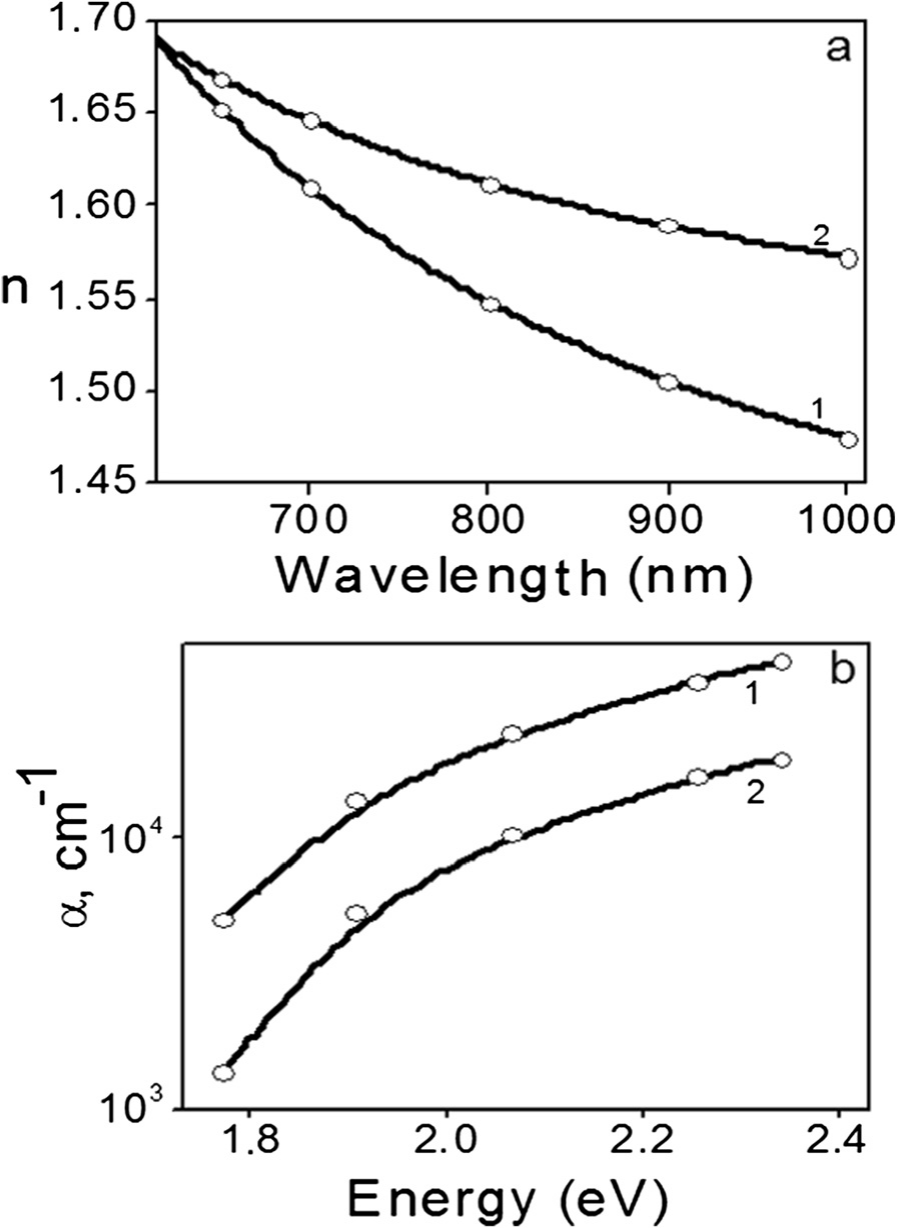
Optical characteristics of spin (1) and dip (2) coating hybrid films: **(a)** refractive index; **(b)** absorption coefficient.

Optical characteristic (refractive index and absorption coefficient) of spin and dip coating films were compared using the envelope method to obtain the optical constants (see Figure 3). Different values of these constants can be evidence of various spatial organizations and directions of the aggregation of dye molecules within the body of hybrid films.

**Figure 4:**
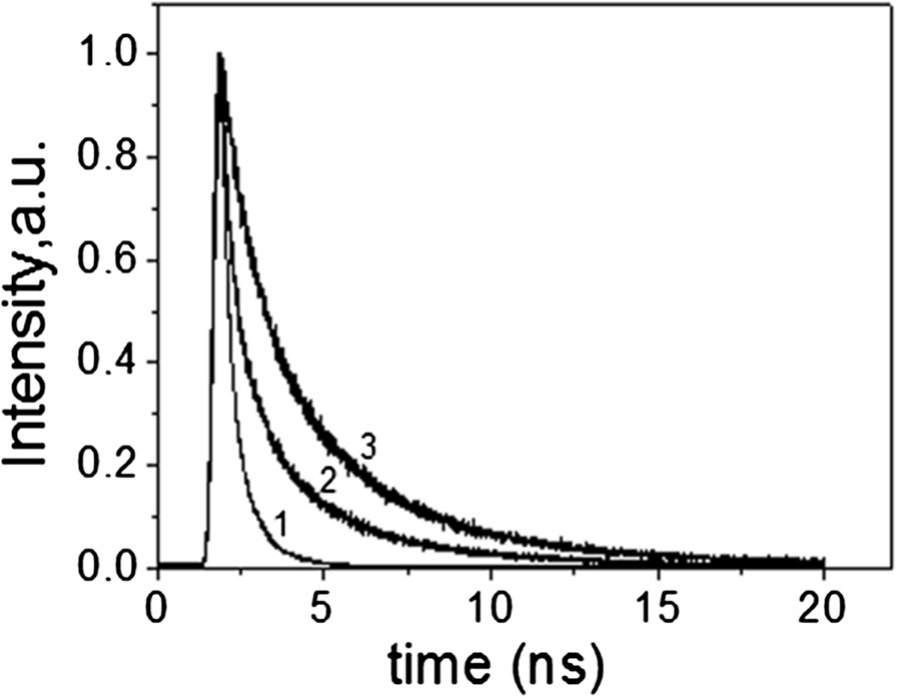
The normalized fluorescence decay profiles for the hybrid film. Concentration Rh6G(mol/l): 1 − 1·10^−5^; 2 − 7.5·10^−3^; 3 − 2.1·10^−2^.

According to [22], in ethanol, the lifetime of Rh6G is reported to be 3.8 ns, with a fluorescence quantum yield of 0.94 as well as good optical and thermal stability; Rh6G has been extensively employed as the lasing medium in liquid optically pumped dye lasers. The presence of H- and J-type aggregates still does not attest to the applicability of these films. Time-resolved fluorescence studies help to show the nature of the aggregates (see Figure 4). The slight increase in the lifetime of the dominant component further supports the above assertion that the excited state is somewhat stabilized through interactions with the Pluronic P123 molecules of the mesostructure (Figure 4). The origin of the minor component cannot be ascertained at this point, but this could be due to the excited-state charge-transfer complex between Rh6G and surrounding Pluronic molecules.

We investigate nonlinear optical properties of hybrid nanocomposites by applying the method of the dynamic holography with nanosecond pump impulses. In all samples indicated in Table 1, we observe the recording of a dynamical grating in the regime of single-impulse pump. The diffraction efficiency was of the order of *η* approximately 0.001 (where *η* is calculated as the intensity ratio between output beam in the first diffraction order and the input beam*η* = *I*_{+1}_/*I*_0_). This value was enhanced in the samples No.1 and No.2 by increasing the concentration of the dye. We can calculate the values of the nonlinear susceptibility of the nanostructured films used by conventional formulas [11,14]:

1 $$ {I}_{\left\{-1\right\}}\left(d,t\right)={I}_{\left\{+1\right\}}\left(d,t\right)=T{I}_0\left(0,t\right)\left[{J}_1^2\left(\delta \right)+{J}_2^2\left(\delta \right)\right] $$

where the argument $\delta =\frac{k_0}{2{n}_0\alpha \cos \phi}\left(1-T\right)\cdot \varDelta n$, *T* is the transmission of the film, *α* is the absorption coefficient, *n*_0_ is the refractive index, *k*_0_ = 2*π*/*λ* is the wavevector, 2φ is the converge angle of pump beams, *Δn* = *n*_2_*I*_0_ is the photoinduced refractive index, where in a Kerr-like medium *n*_2_ denotes the coefficient of the nonlinear refraction.

From (1) we obtain the formula, from which we can calculate the value *n*_2_:

2 $$ \eta \approx \frac{\pi }{n_0 \cos \kern.5em \%\pi}\frac{T\left(1-T\right)}{\alpha}\cdot {n}_2{I}_0 $$

From (2): *n*_2_ = 1.5⋅10^−8^ cm^2^/W for spincoating films (the thickness *d* = 200 nm) and *n*_2_ = 3.9⋅10^−9^ cm^2^/W for dipcoating films (the thickness *d* = 800 nm). The nonlinear optical susceptibility is calculated from the following expression:

3 $$ {\chi}^{(3)}\left[ esu\right]={n}_2\left[\frac{c{m}^2}{W}\right]\cdot \frac{9\cdot {10}^4}{4\pi }c{\varepsilon}_0{n}_0^2 $$

where *c* is the light velocity in vacuum, *ε*_0_ is the dielectric constant. We obtain that the nonlinear susceptibility is in the range *χ*^(3)^ = 8.6⋅10^−7^ esu and *χ*^(3)^ = 2.2⋅10^−7^ esu for spin and dipcoating films, respectively. This giant value of *χ*^(3)^ is the result of a rather high optical density obtained in the films with very thin thickness.

**Figure 5:**
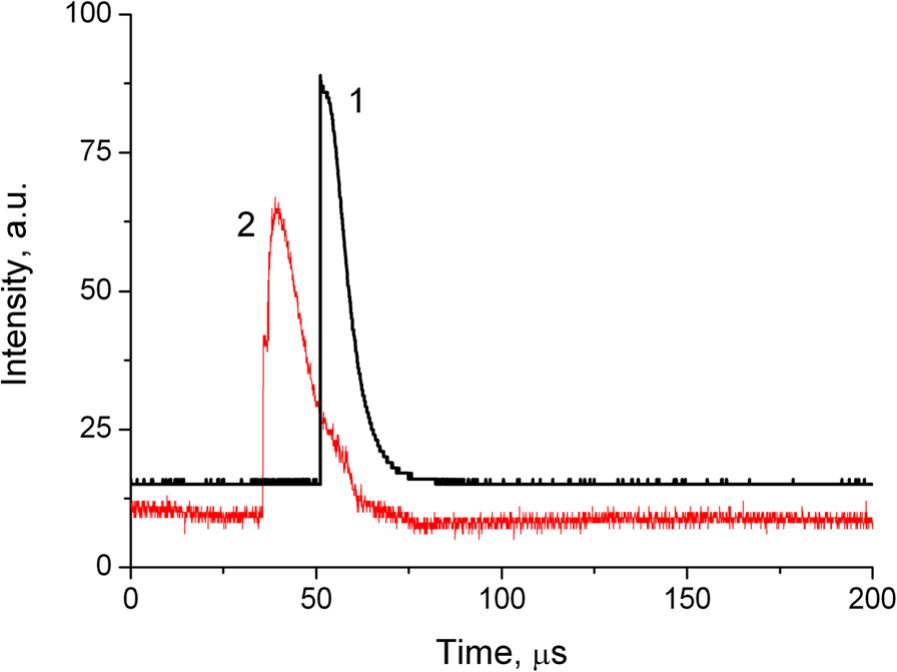
Oscillogram of output intensities in first diffraction orders: 1 - probe beam, 2 - self-diffracted beam.

A typical oscillogram of the output intensity in the first diffraction order of the probe beam after the action of one pump impulse is shown in Figure 5, curve 1. For comparison, the oscillogram of the input pulse is presented as well (Figure 5, curve 2). One can see that the relaxation process of the recording grating is almost the same as the pumping pulse that proves the dynamical nature of the grating. We suppose the nonlinear mechanism of the grating recording is the electron nonlinearity in dye molecule Rh6G under excitation of laser radiation.

But we should note that the investigated films experienced degradation after the action of a series of pulses. We suppose a relief grating is created due to the ablation on the surface of the film. Then, we observe the recording of a stationary phase grating with many diffraction orders for a probe beam. In this case, the diffraction efficiency of the stationary grating is increasing and remains almost the same in all diffraction orders. The rigid relief of the grating obtained on the nanostructured films may be considered for creation of miniature beam multiplexer. Besides, the space period of such grating can be significantly decreased in the silicon nanostructures that lead to greatly increase of the numbers of the diffraction orders.

## Conclusions

The ability of ultrafast excited-state dynamics of composite films containing Rh6G was examined for their potential use as the photonic layer in an all-optical switching device. The derived sol-gel composite films consisted of a silica framework and self-assembled organic mesostructures formed by the nonionic surfactant Pluronic P123. Obtained films showed remarkable physical and optical properties. The excellent processability of the precursor sol made it possible to fabricate high-quality films, of varying thicknesses, using dip or spincoating. The ability of the occluded Pluronic P123 mesostructures to solubilize organic molecules made these films ideal host matrices for organic dyes and molecular assemblies with substantial nonlinear susceptibilities due to exiton dynamics in film structure.

We have obtained the recording of a dynamical grating in a single-pulse regime. Laser pulses of the second harmonic Nd^3+^ laser of nanosecond duration are used. A giant value of the nonlinear optical susceptibility was obtained experimentally (*χ*^(3)^ = 8.6⋅10^−7^ esu and *χ*^(3)^ = 2.2⋅10^−7^ esu for spin and dipcoating films, respectively) in thin nanoscale photosensitive films. Since the dynamical grating exhibits the fastest relaxation time (up to 10 ns), the nonlinear mechanism represents an electronic excitation of the photosensitive molecules. As far as the dye molecules are distributed in nanoporous silica, a model of ‘gas of molecular dye’ may be rightly used in order to consider nonlinear optical properties in the nanostructured hybrid films. We suppose that further improvement of the nonlinear optical nanomaterials may follow on the way to embed additional inclusions, which will not promote the heat accumulation in the host matrix and will lead to effective dissipation of the heat energy. We are currently working in this direction.
